# Atlantic Salmon Pre-smolt Survivors of *Renibacterium salmoninarum* Infection Show Inhibited Cell-Mediated Adaptive Immune Response and a Higher Risk of Death During the Late Stage of Infection at Lower Water Temperatures

**DOI:** 10.3389/fimmu.2020.01378

**Published:** 2020-06-30

**Authors:** Marco Rozas-Serri, Carlos Lobos, Rodolfo Correa, Ricardo Ildefonso, Jorge Vásquez, Ariel Muñoz, Lucerina Maldonado, Victoria Jaramillo, Darling Coñuecar, Camila Oyarzún, Romina Walker, Carolina Navarrete, Jorge Gayosa, Patricio Mancilla, Andrea Peña, Carolina Senn, Francisco Schwerter

**Affiliations:** ^1^Laboratorio Pathovet Ltda., Puerto Montt, Chile; ^2^Newenko Group SpA., Puerto Montt, Chile; ^3^Hendrix Genetics Aquaculture S.A., Puerto Varas, Chile

**Keywords:** *Renibacterium salmoninarum*, BKD, cell-mediated, immune response, water temperature

## Abstract

Bacterial kidney disease (BKD) is widespread in many areas of the world and can cause substantial economic losses for the salmon aquaculture industry. The objective of this study was to investigate the pathophysiological response and gene expression profiles related to the immune response at different water temperatures and to identify the best immunopathological biomarkers to define a phenotype of resistance to BKD. The abundance of *msa* transcripts of *R. salmoninarum* in the head kidney was significantly higher in infected fish at 11°C. *R. salmoninarum* induced significantly more severe kidney lesions, anemia and impaired renal function at 11°C. In addition, the expression pattern of the genes related to humoral and cell-mediated immune responses in infected fish at 11 and 15°C was very similar, although *R. salmoninarum* induced a significantly greater downregulation of the adaptive immune response genes at the lower water temperature. These results could be due to a suppressed host response directly related to the lowest water temperature and/or associated with a delayed host response related to the lowest water temperature. Although no significant differences in survival rate were observed, fish infected at the lowest temperature showed a higher probability of death and delayed the mortality curve during the late stage of infection (35 days after infection). Thirty-three immunopathological biomarkers were identified for potential use in the search for a resistance phenotype for BKD, and eight were genes related specifically to the adaptive cell-mediated immune response.

## Introduction

Bacterial kidney disease (BKD) or Renibacteriosis is caused by the facultative intracellular bacteria *Renibacterium salmoninarum*, which is widespread in many areas of the world and can cause substantial economic losses for the salmon aquaculture industry (1, 2). *R. salmoninarum* infection is characterized by chronic disease progression and infects both freshwater and saltwater salmonid life stages (3). Chronic BKD is associated with granulomatous lesions and white to gray-white abscesses in internal organs, such as the kidney (4, 5). Periods of stress, such as during smoltification or environmental change, can increase the disease severity and clinical signs (3). Although vaccines against the bacterium exist, BKD is still prevalent in sea cages due to poor efficacy of vaccines and antibiotics (6).

*Renibacterium salmoninarum* has been shown to be highly clonal with limited phenotypic and genotypic variation (7–11). However, whole-genome single-nucleotide polymorphism-based comparisons identified a deep phylogenetic division of *R. salmoninarum* in the population, which provides evidence for the transatlantic transmission and spread over decadal scales (1). Specific to Chile, multiple introductions of *R. salmoninarum* into the country from global sources over 30 years have been reported based on whole-genome sequencing (2).

Water temperature is one of the most important factors that influence the dynamics of BKD because it strongly regulates the replication rate of the bacteria and the immune response of the fish (12). Fish infected with *R. salmoninarum* can survive and even recover, although whether infected fish can completely eliminate the infection is unknown (12–14). Some studies indicate that higher water temperatures increase BKD mortality (13), while other studies have reported the opposite (12, 15). In addition to the effect of temperature on pathogen replication and host immunity, changes in temperature are an important source of stress that can alter the pathogen-host interaction (12). In addition, *R. salmoninarum* infections are complicated and mortality is only one of the possible outcomes of a chronic infection (12). How *R. salmoninarum* modulates the fish's immune response and what factors contribute to the immunopathology of the disease remain poorly understood.

The systemic nature of the BKD infection has been demonstrated, and the host cytokine and cytokine-related genes are affected during the early stage of *R. salmoninarum* infection in rainbow trout (*Oncorhynchus mykiss*) (16) and Chinook salmon (*Oncorhynchus tshawytscha*) (17). Some studies have examined immune gene expression changes early after *R. salmoninarum* infection or after vaccination and demonstrated the differential regulation of key immune genes related to inflammatory response (16–20). Eslamloo et al. (20) showed that formalin-killed *R. salmoninarum* induced the expression of genes associated with inflammation and cytokine responses but suppressed the expression of genes that have putative roles as a cytokine receptor and kinase regulator. However, no information is available regarding the immunopathological and cell-mediated immune response during the late stage of infection in Atlantic salmon (Salmo salar) kept at different water temperatures (11 and 15°C).

To investigate the late stage of the interaction of *R. salmoninarum* with infected surviving fish, we studied the pathology and kinetics of gene expression related to the innate and adaptive immune response to identify and select the best and most predictive immunopathological biomarkers, with the goal of defining more robust phenotypes of resistance of Atlantic salmon to BKD.

## Materials and Methods

### *Renibacterium salmoninarum* Isolate

An *R. salmoninarum* strain (Rs2) previously isolated from a commercial farm located in the Magallanes Region of Chile and belonging to Elanco was used. The Rs2 isolate was grown in SKDM medium at 15°C ± 2°C for 15 days. Bacterial colonies were collected from the plates and suspended in 1 ml of 0.9% saline solution. The absorbance at 625 nm (DO_625_) was measured using a spectrophotometer (Biobase Brand Model BK-UV1000) to quantify the biomass produced. Ten milliliters of inoculum of Rs2 with an OD_625_ of 1.0 each was obtained. This OD_625_ corresponded to approximately 6.84 × 10^6^ u.f.c./mL.

### *Renibacterium salmoninarum* Challenge

The challenge was carried out at the Elanco Animal Health experimental hatchery (Ruta 5 Sur, Km 1012, Puerto Varas, Chile). Non-vaccinated Atlantic salmon, *Salmo salar* L., fry (Hendrix Genetics Aquaculture Chile), with an average size of 25 g, were used for the challenge. Prior to challenge, 30 fish were randomly tested and screened to ensure that they were enzootic pathogen-free using PCR for piscine reovirus (PRv), infectious pancreatic necrosis virus (IPNv), infectious salmon anemia virus (ISAv), *Flavobacterium psychrophilum* and *R. salmoninarum* (21–25). Kidneys from these fish were also histologically examined for microscopic lesions before the challenge.

Two Atlantic salmon stocks were acclimatized at 11 and 15°C for 2 weeks. Then, an intraperitoneal (i.p.) challenge was conducted in two identical independent systems, each with four 100-liter water tanks containing freshwater in which the water flow was regulated to 1.5 changes per hour (Lh^−1^). All fish were identified using pit-tags and distributed at each treatment randomly. Each system considered three tanks (replicates) with 50 fish each injected by i.p. (0.1 mL) and a control tank with 50 fish inoculated with 0.1 mL of sterile saline (0.9%). Infected fish were kept at 11 and 15°C in the first and second system, respectively.

The fish were fed daily with commercial food. The water temperature, dissolved oxygen concentration, feeding and mortality were recorded daily. The experiment was performed over 55 days, and tissue and blood samples were taken from the surviving fish in each tank. In addition, the weight and length of each fish were recorded at the beginning and end of the study period to calculate the condition factor (k), the specific growth rate (SGR) and the thermal growth coefficient (TGC). Elanco's Animal Welfare Program covers all of Elanco's animal facilities worldwide and ensures the care and use of all animals. Therefore, the study that was completed at the Aquarium Facility in Puerto Varas was reviewed and approved by the Elanco Global Ethical Committee (Institutional Animal Care and Use Committee). All efforts were made to provide the best growing conditions and minimize suffering.

### Hematological and Biochemical Blood Profile

Whole blood samples were obtained from the caudal vein of each surviving fish and added to 1.5 mL heparin-lithium tubes. One part of each sample was used to perform a complete blood count test or hemogram, and the rest was centrifuged at 5,000 RPM for 5 min to obtain plasma. The concentrations of substrates, enzymes, electrolytes and minerals in the plasma were quantified using photometric, kinetic and colorimetric methods (Hitachi Cobas c311, Roche Diagnostics, Mannheim, Germany).

### Histopathological Examination

Samples at 0.5 to 1 cm^3^ in volume were collected from the mid-kidney and placed in 10% formalin buffer for at least 24 h. The samples were then dehydrated in a graded alcohol series and processed via a standard histological examination. Sections at 3 μm thick from each tissue were stained with hematoxylin and eosin (H&E) and analyzed by optical microscopy (Leica DM-2000, Hamburg, Germany) using the Leica Application Suite Software (LAS), Image Analysis (Leica, Hamburg, Germany) and a digital camera (Leica DFC-295, Hamburg, Germany). To provide a more unbiased analysis, a semi-quantitative indicator of tissue damage was developed. The BKD histoscore (hsBKD) considered lesions in the kidney according to the criteria described in Table 1. The hsBKD of each fish at the end-time sampling point was used to calculate the average histoscore ± standard deviation.

**Table 1:** Histopathological criteria and semi-quantitative weighting used to define hsBKD in the kidney.

**Histoscore**	**HT hyperplasia**	**MMC-hyperplasia**	**Granulomas**	**Rs-like**	**Proliferative GNP**
0	No alteration	No alteration	Without granulomas	No bacteria	No alteration
0.1–0.99	Mild hyperplasia (≤10% tissue surface)	Mild hyperplasia (≤10% tissue surface)	≤10% tissue surface	Focal presence, slightly noticeable	Parcial, focal mesangial proliferation
1.0–1.99	Moderate hyperplasia (>10% ≤50% tissue surface)	Moderate hyperplasia (>10% ≤50% tissue surface)	>10% ≤50% tissue surface	Focal presence, very evident	Global, focal mesangial proliferation
2.0–3.0	Severe hyperplasia (>50% tissue surface)	Severe hyperplasia (>50% tissue surface)	>50% tissue surface	Diffuse presence	Global, diffuse mesangial proliferation
Relative ponderation	0.09	0.09	0.54	0.18	0.1

*The scoring system considers the mean of the observations of five non-overlapping high-magnitude optical fields (HMOFs, 20X) per kidney sample. GNP: glomerulonephritis; MMC: melano-macrophage centers; HT, hematopoietic tissue; Rs-like, bacteria similar to R. salmoninarum*.

### RNA Extraction

Samples at 0.5 cm^3^ in volume were obtained from the head kidney, placed in tubes with RNAlater® and stored at −80°C until further analysis. All tissue samples were placed in microtubes with 1 mL TRIzol and ceramic beads and homogenized in a BeadBug® Microtube Homogenizer (Benchmark Scientific, Edison, NJ, USA) at room temperature. Then, 200 μL of chloroform:isoamyl alcohol was added, vigorously mixed and allowed to stand for 2 min before centrifuging at 4°C for 15 min at 12,000 G. The supernatant was transferred to a new tube and mixed with 400 μL of 70% ethanol. This mixture was passed through the columns with the E.Z.N.A.® Tissue RNA Kit (Omega Bio-Tek Inc., Norcross, GA, USA) according to the manufacturer's instructions for RNA extraction. Total RNA was quantified using the fluorimetry method in a Qubit^TM^ 3.0 Fluorometer (Invitrogen^TM^, Thermo Fisher Scientific, Wilmington, DE, USA), and the quality of the total RNA was determined by visualizing the RNA bands separated by electrophoresis in 1% agarose gels using a FlashGel^TM^ System (Lonza Group, Allendale, NJ, USA).

### Abundance of msa Transcripts of *R. salmoninarum*

The abundance of msa transcripts was determined using RT-qPCR in a StepOnePlus^TM^ Real-Time PCR system (Applied Biosystems, Life Technologies, Waltham, MA, USA) with the Brilliant III Ultrafast RT-qPCR Master Mix kit (Agilent Technologies, Santa Clara, CA, USA). Relative quantification of mRNA of the major soluble antigen (*msa*, p57) gene of *R. salmoninarum* was performed using RT-qPCR TaqMan® as described in Suzuki and Sakai (23). The amplification was performed in a final volume of 15 μL containing 300 nM primers, 400 nM probe, 300 nM ROX (50 nM) and 100 ng total RNA. The RT-qPCR program consisted of a reverse transcription step at 50°C for 10 min, followed by 3 min of activation and denaturation at 95°C, and 45 cycles of 15 s at 95°C and 30 s at 60°C. The abundance of mRNA transcribed from the *msa* gene mRNA of *R. salmoninarum* is expressed as the relative number of copies of the gene in infected fish compared to the number of copies in the uninfected control group and was calculated as 2^(CTuninfectedfish−CTinfectedfish)^. Relative values of the *msa* transcripts of *R. salmoninarum* were expressed as log_10_ fold (log fold).

### RT-qPCR for Immune Response Genes

The RNA extraction and relative quantification of the immune related genes was evaluated in the head kidney of fish at the end-time sampling point by normalized RT-qPCR as described by Rozas-Serri et al. (26, 27) (Table 2). Briefly, differential expression of selected genes was determined with the Real-Time PCR StepOnePlus^TM^ system (Applied Biosystems, Life Technologies, Waltham, MA, USA) using the Brilliant II SYBR Green qPCR Master Mix kit (Agilent Technologies, Santa Clara, CA, USA). Each amplification reaction was performed in a final volume of 15 μl, which consisted of 7.5 μl of buffer, 250 nM to 750 nM primers depending on the gene, 300 nM ROX (50 nM) and 2 μl of cDNA diluted 1:10.

**Table 2:** Genes, primers, efficiency, correlation coefficients, and optimal annealing temperatures for the reference and target genes.

**Gene name**	**Primers sequence (5' → 3')**	**Accesion number**	**Tm (^**°**^C)**	**Efficiency**	***R*^**2**^**
*ifng*	CTAAAGAAGGACAACCGCAG	AY795563	56	1.95	0.995
	CACCGTTAGAGGGAGAAATG				
*ifga*	TGCAGTATGCAGAGCGTGTG	DQ354152	60	1.91	0.999
	TCTCCTCCCATCTGGTCCAG				
*il1b*	ATCACCATGCGTCACATTGC	NM_001123582	58	2.05	0.997
	GTCCTTGAACTCGGTTCCCA				
*il2*	CATGTCCAGATTCAGTCTTCTATACACC	AM422779	60	1.95	0.998
	GAAGTGTCCGTTGTGCTGTTCTC				
*il4/13*	ACCACCACAAAGTGCAAGGAGTTC	FN820501	60	1.89	0.999
	CACCTGGTCTTGGCTCTTCACAAC				
*il8*	GGCCCTCCTGACCATTACT	NM_001140710	56	2.01	0.997
	ATGAGTCTACCAATTCGTCTGC				
*il10*	CGCTATGGACAGCATCCT	EF165029	55	2	0.998
	AAGTGGTTGTTCTGCGTT				
*il12b*	CTGAATGAGGTGGACTGGTATG	BT049114	55	2.1	0.999
	ATCGTCCTGTTCCTCCG				
*il17*	TGGTTGTGTGCTGTGTGTCTATGC	GW574233	60	1.92	0.99
	TTTCCCTCTGATTCCTCTGTGGG				
*tgfβ*	AGTTGCCTTGTGATTGTGGGA	EU082211	60	2.04	0.996
	CTCTTCAGTAGTGGTTTGTCG				
*mhc1*	CTGCATTGAGTGGCTGAAGA	AF508864	60	1.99	0.998
	GGTGATCTTGTCCGTCTTTC				
*mhc2*	TCTCCAGTCTGCCCTTCACC	BT049430	60	2.03	0.996
	GAACACAGCAGGACCCACAC				
*cd4*	GAGTACACCTGCGCTGTGGAAT	NM_001124539	60	2.01	0.973
	GGTTGACCTCCTGACCTACAAAGG				
*cd8b*	CGCACACACCTCAACAACTC	AY693394	56	1.94	0.945
	ATTGATGCGCAGTGTGAAAG				
*igm*	TCTGGGTTGCATTGCCACTG	CA039888	60	2.09	0.998
	GTAGCTTCCACTGGTTTGGAC				
*igt*	CAACACTGACTGGAACAACAAGGT	GQ907004	60	2.05	0.998
	CGTCAGCGGTTCTGTTTTGGA				
*gata3*	CCCAAGCGACGACTGTCT	EU418015	60	1.90	0.999
	TCGTTTGACAGTTTGCACATGATG				
*stat1*	GAACATGGAGGAGTCCAATGGAAGC	CA343225	60	1.93	0.990
	GGACCCTCATTTGATCTGTTGCCT				
*eomes*	ACCTCTCGTCGTCAGATACTG	EU418014	56	1.98	0.999
	GGACCGGTGAGTCTTTTCTTC				
*tbx21*	GGTAACATGCCAGGGAACAGGA	FM863825	58	2.1	0.999
	TGGTCTATTTTTAGCTGGGTGATGTCTG				
*gzma*	GACATCATGCTGCTGAAGTTG	BT046910	60	1.9	0.992
	TGCCACAGGGACAGGTAACG				
*mpeg1*	GGCAACATCACCTACTCCATAA	XM_014172502	60	2.09	0.999
	AGGTTGTTCTTGGTGCTCTC				
*β-actina*	ACGAGAGGTTCCGTTGTCC	BG933897	60	2.1	0.999
	GCAAGACTCCATACCGAGGA				
*ELF-1α*	CCCCTCCAGGACGTTTACAAA	NM_001123629	60	2	0.997
	CACACGGCCCACAGGTACA				

The PCR program consisted of a 10-min activation and denaturation step at 95°C, followed by 45 cycles of 15 s at 95°C, 30 s at the annealing temperature of the corresponding primers and an additional 15 s extension at 72°C. Five biological replicates were used, and each qPCR reaction was run in duplicate, including a negative control without reverse transcriptase to check for genomic DNA contamination and a negative control without template to check for primer dimers. The relative expression results were analyzed using amplification efficiencies as described by Pfaffl et al. (28). *ELF1A* and ß*-actin* were selected as housekeeping genes for gene normalization as described by Rozas-Serri et al. (26).

### Statistical Analysis

A total of 400 individuals were randomly assigned to the eight experimental groups. The mortality analysis was performed using descriptive statistics and hypothesis testing. Differences in the cumulative mortality rate between groups and their replicates were determined using the chi-squared test, and differences in the meantime to mortality were analyzed using the Mann-Whitney U-test. The cumulative survival function [S(t)] was estimated with the Kaplan-Meier method (29) and separately for each water temperature. Significant differences in S(t) were determined using a Cox proportional hazards model (CPHM). The level of significance was established at *p* < 0.05.

Each variable was descriptively explored based on the time point and water temperature. The level of significance was set at *p* < 0.05. Each variable, including water temperature, was analyzed by an analysis of variance. The association degree between the variables was determined by a correlation matrix, while the dependence degree of each immunopathological variable with the abundance of msa transcripts was explored using simple linear regressions. All analyses were performed using the statistical package Stata, version 13 (StataCorp LP, College Station, TX, USA). Finally, non-parametric principal coordinate analysis (PCO) was used to assess the multivariate association of different variables from the fish response. Euclidean distance matrices for each group of variables and the data set were used. The PCO analysis was performed with the statistical package PRIMER, version 6 (PRIMER-e, Auckland, New Zealand).

## Results

### Mean Survival Rates at the Different Water Temperatures Did Not Differ, Although the Probability of Death Was Higher, and the Mortality Curve Was Delayed in Infected Fish at Lower Temperatures

**Figure 1:**
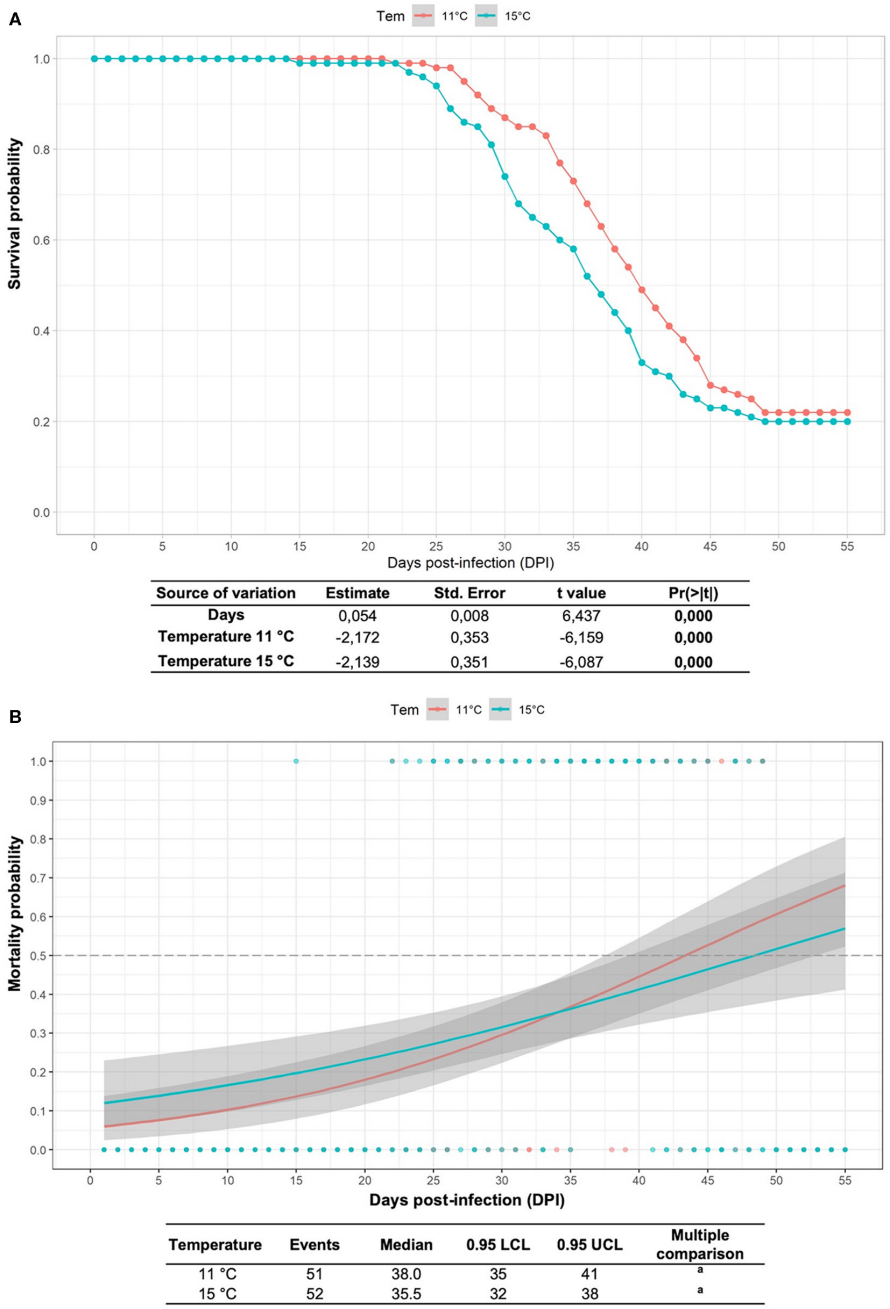
Survival study and mortality probability model in Atlantic salmon pre-smolts infected with *R. salmoninarum*. **(A)** Kaplan-Meier estimator of the cumulative survival function for fish infected with *R. salmoninarum* at 11 and 15°C considering three replicates. The green and red shaded area represents the 95% confidence interval. **(B)** Multiple comparison logRank test for mortality probability. The gray shaded area represents the 95% confidence interval. Tem, temperature.

The cumulative mortalities and survival study results of fish infected with *R. salmoninarum* and kept at different water temperatures are shown in Figure 1. In the group infected at 15°C, the onset of mortality occurred at 15 dpi and the mean days to death was 35.5 dpi. In the stock infected at 11°C, the onset of mortality occurred at 22 dpi and the mean days to death was 38 dpi. The mean survival rates did not differ between infected fish at 11°C (21.5%) and 15°C (21.1%), although a higher probability of death was observed in infected fish at 11°C than at 15°C after 35 days postinfection (Figure 1). No mortality was observed in the control group.

### Abundance of msa Transcripts of *R. salmoninarum* in the Kidney Is Significantly Higher in Surviving Fish Exposed to Lower Water Temperatures

**Figure 2:**
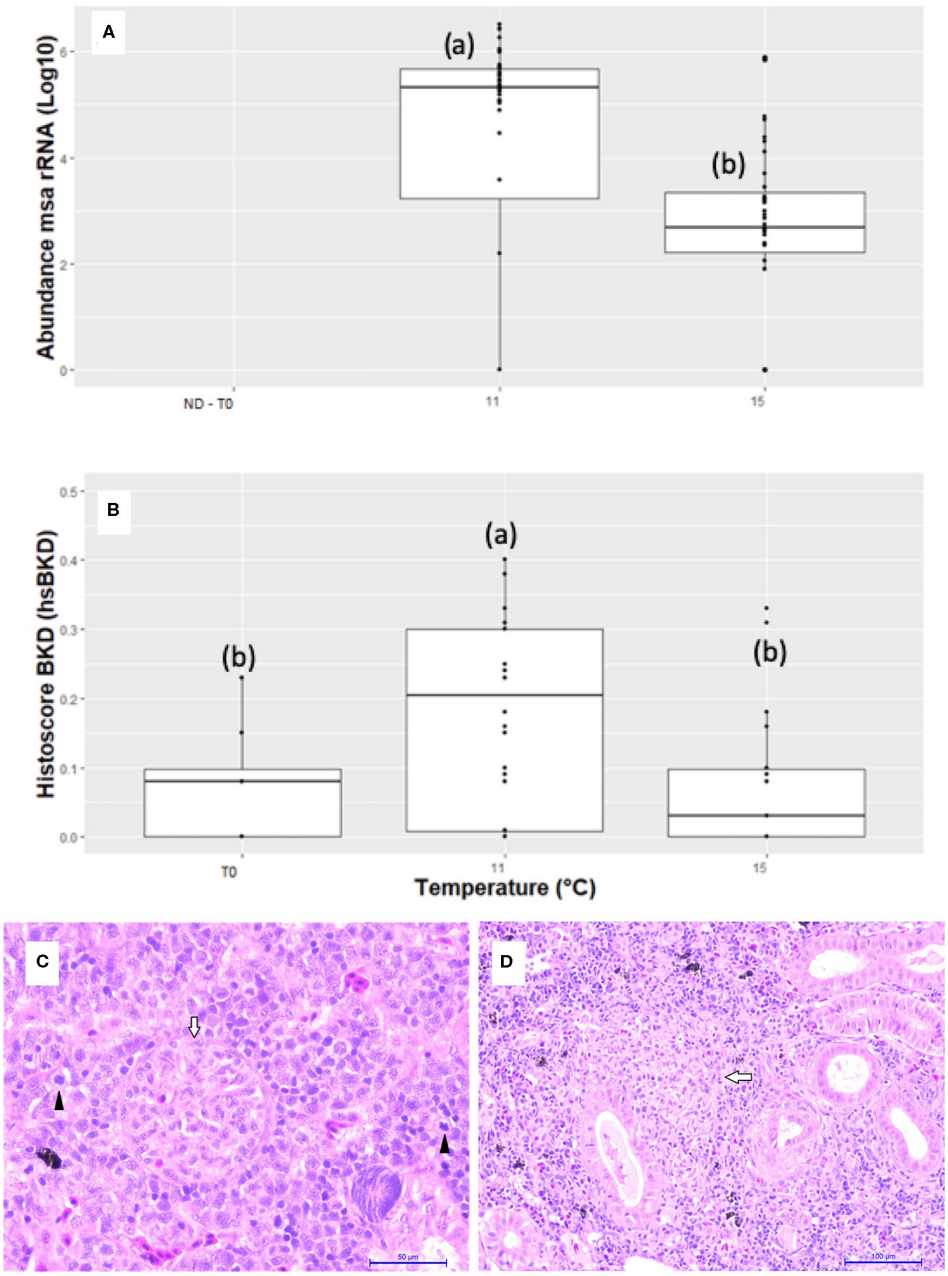
Abundance of msa transcripts of *R. salmoninarum* and BKD histoscore in the head kidneys of Atlantic salmon pre-smolts infected at 11 and 15°C. **(A)** The abundance of msa transcripts of *R. salmoninarum* in infected fish at 11 and 15°C and non-infected fish. The relative quantification of the msa gene in kidneys is expressed as the log_10_ value of the number of copies of the msa gene of R. salmoninarum in the infected fish group compared to that in the non-infected control group (ND, not detected). **(B)** Histopathological lesions expressed as a BKD histoscore in infected fish at 11 and 15°C and non-infected fish. **(C)** Hyperplasia with mitotic figures (black arrows) in hematopoietic tissue, renal corpuscle with mesangial cell hyperplasia (white arrow) and capillary obliteration (HandE, 40X bar 50 μm). **(D)** Mixed granuloma in the initial stage of formation (white arrow) in kidney of Atlantic salmon pre-smolts infected with *R. salmoninarum* (HandE, 20X bar 100 μm).

Infected fish at 11°C showed a significantly higher amount of *msa* transcripts of *R. salmoninarum* in the kidney than infected fish at 15°C at 55 dpi (Figure 2). However, water temperature could have modulated the abundance of msa transcripts by changing the magnitude and/or timing of the host response. Thirty-three immunopathological biomarkers were significantly associated with the abundance of *msa* transcripts of *R. salmoninarum* at different water temperatures (Table 3). The individual values of the biomarkers analyzed in this study are presented in Supplementary Table 1.

**Table 3:** Goodness-of-fit test results for a simple linear regression between the immunopathological outcomes of fish challenged by *R. salmoninarum* at different water temperatures.

**Biomarker group**	**Abbreviation**	**Biomarker**	**Df**	**SS**	**MS**	***F*-value**	**Pr(>F)**	***R*^**2**^**	**R-adjusted**
Microscopic lesions	hsBKD	Histoscore BKD	1	0.26927	0.26927	55.886	0.00000	0.381	0.374
Plasma enzymes. substrates and minerals profile	Sodium (mmol/L)	NA	1	1642.8	1642.82	29.712	0.00000	0.287	0.277
	Chloride (mmol/L)	CL	1	650.5	650.47	13.414	0.00047	0.155	0.144
	Potassium (mmol/L)	K	1	0.7644	0.76439	11.268	0.00125	0.132	0.120
	Phosphorus (mmol/L)	P	1	381915	381915	295.54	0.00000	0.846	0.843
	Iron (mmol/L)	FE	1	577417	577417	124.04	0.00000	0.646	0.641
	Magnesium (mmol/L)	MG	1	674.67	674.67	62.068	0.00000	0.466	0.459
	Calcium (mmol/L)	CA	1	1084.9	1084.92	30.556	0.00000	0.301	0.291
	Alcaline phosphatase (U/L)	ALP	1	331208	331208	392.22	0.00000	0.875	0.873
	Pancreatic amilase (U/L)	PAM	1	−1.2237	0.1297	−9.435	0.00000	0.655	0.647
	Lipase (U/L)	LIP	1	−0.23511	0.02741	−8.579	0.00000	0.610	0.602
	Total amilase (U/L)	AMI	1	−1.2319	0.1492	−8.259	0.00000	0.563	0.555
	Creatine kinase (U/L)	CK	1	34.354	34.354	38.502	0.00000	0.407	0.397
	Aspartate aminotransferase (U/L)	AST	1	124928	124928	12.914	0.00065	0.175	0.161
	Alaline aminotransferase (U/L)	ALT	1	0.2764	0.276398	2.8226	0.09823	0.046	0.029
	Lactate dehydrogenase (U/L)	LDH	1	0.0884	0.08843	0.2331	0.63150	0.005	−0.016
	Cholesterol (mmol/L)	CHOL	1	979.86	979.86	391.12	0.00000	0.827	0.825
	Albumin (g/L)	ALB	1	9.096	9.096	126	0.00000	0.612	0.607
	High density lipoprotein (mmol/L)	HDL	1	133.24	133.24	94.498	0.00000	0.578	0.572
	Urea (mmol/L)	URE	1	107.378	107.378	114.53	0.00000	0.566	0.561
	Low density lipoprotein (mmol/L)	LDL	1	22.697	22.6965	57.944	0.00000	0.457	0.449
	Total protein (g/L)	TPO	1	7.4125	7.4125	70.242	0.00000	0.436	0.429
	Glucose (mmol/L)	GLU	1	13.328	13.3282	57.934	0.00000	0.429	0.422
	Globulins (g/L)	GLO	1	5.5818	5.5818	49.639	0.00000	0.353	0.346
	Lactate (mmol/L)	LAC	1	0.47979	0.47979	18.456	0.00004	0.169	0.160
	Uric acid (μmol/l)	UAC	1	1191	1191.04	17.006	0.00008	0.158	0.148
	Creatinine (μmol/l)	CRE	1	1677.4	1677.38	13.779	0.00037	0.144	0.133
	Triglycerides (mmol/L)	TRG	1	0.0002	0.000201	0.0046	0.94640	0.050	−0.011
Hematological profile	Hematocrit (%)	HCT	1	11.6577	11.6577	231.53	0.00000	0.773	0.770
	Red blood cell count (x10e6/UI)	RBC	1	6.6981	6.6981	221.44	0.00000	0.735	0.731
	Hemoglobin (g/L)	HB	1	7.0518	7.0518	67.127	0.00000	0.438	0.432
	Mean Corpuscular Hemoglobin Concentration (g/L)	MCHC	1	0.37926	0.37926	14.353	0.00028	0.143	0.133
	Mean Corpuscular Hemoglobin (f/L)	MHC	1	0.0439	0.04391	0.9586	0.33050	0.012	−0.001
	Immature red blood cell (N°/mL)	IRBC	1	20175918	20175918	67.026	0.00000	0.626	0.617
	Lymphocytes count (N°/mL)	LYM	1	814043587	814043587	63.828	0.00000	0.477	0.470
	Heterophils count (N°/mL)	HET	1	13530470	13530470	54.819	0.00000	0.450	0.442
	Monocytes count (N°/mL)	MON	1	124.54	124.544	34.336	0.00000	0.389	0.377
	White blood cell count (N°/μL)	WBC	1	739782809	739782809	42.168	0.00000	0.376	0.367
	Blastocytes (N°/mL)	BLA	1	452070	452070	18.157	0.00009	0.275	0.259
	Thrombocytes or Platelet Count (N°/μL)	PLC	1	2.9866	2.98657	11.172	0.00191	0.232	0.211
	Unidentified cells (N°/mL)	UIC	1	196	195.99	1.9546	0.17040	0.050	0.025
Immunological profile (RT–qPCR)	Cluster of differentiation 8	CD8	1	10.9629	10.9629	155.27	0.00000	0.680	0.676
	Eomesodermin	EOMES	1	7.4297	7.4297	113.36	0.00000	0.669	0.663
	Interleukin 12	IL-12	1	6.4925	6.4925	116.23	0.00000	0.663	0.658
	Interleukin 2	IL-2	1	2.561	2.56097	61.368	0.00000	0.600	0.590
	T-bet	TBET	1	2.9251	2.92511	87.005	0.00000	0.596	0.589
	Granzyme A	GZMA	1	23.142	23.1419	49.221	0.00000	0.468	0.458
	Perforin 2	MPEG	1	2.4807	2.48073	38.634	0.00000	0.408	0.398
	Interleukin 10	IL-10	1	18.428	18.4285	38.98	0.00000	0.386	0.376
	Immunoglobulin T	IGT	1	6.058	6.058	33.35	0.00000	0.282	0.273
	GATA-3	GATA3	1	3.6406	3.6406	23.095	0.00001	0.281	0.269
	Interferon gamma	IFNG	1	1.785	1.785	15.894	0.00025	0.265	0.249
	Interleukin 4/13	IL4/13	1	0.85802	0.85802	20.802	0.00003	0.261	0.248
	Interleukin 17	IL-17	1	0.078697	0.078697	11.834	0.00129	0.212	0.194
	Immunoglobulin M	IGM	1	2.155	2.15496	21.669	0.00001	0.192	0.184
	Interleukin 8	IL8	1	0.7799	0.77992	13.9	0.00042	0.183	0.170
	Transforming growth factor beta	TGFB	1	0.37021	0.37021	10.454	0.00201	0.151	0.136
	Interleulin 1 beta	IL-1B	1	0.4017	0.40172	3.5993	0.06270	0.058	0.042
	Cluster of differentiation 4	CD4	1	0.1036	0.103601	2.2298	0.13920	0.026	0.015
	STAT1	STAT1	1	0.1424	0.14238	0.7875	0.37850	0.013	−0.004
	Interferon alpha	IFNA	1	0.0231	0.023098	0.7709	0.38280	0.010	−0.003
	Type I histocompatibility complex	MHCI	1	0.0691	0.069117	0.6599	0.41920	0.009	−0.005
	Type II histocompatibility complex	MHCII	1	0.00711	0.007109	0.1855	0.66800	0.003	−0.011

*In black, biomarkers that showed a significant degree of association (p < 0.0001; R^2^ > 0,3) with the abundance of msa transcription of the bacterium in Log_10_ in kidney samples (Df, degree of freedom; SS, sum of squares; MS, mean squares)*.

### *R. salmoninarum* Induces More Severe Kidney Damage in Surviving Fish Exposed to Lower Water Temperatures

**Figure 3:**
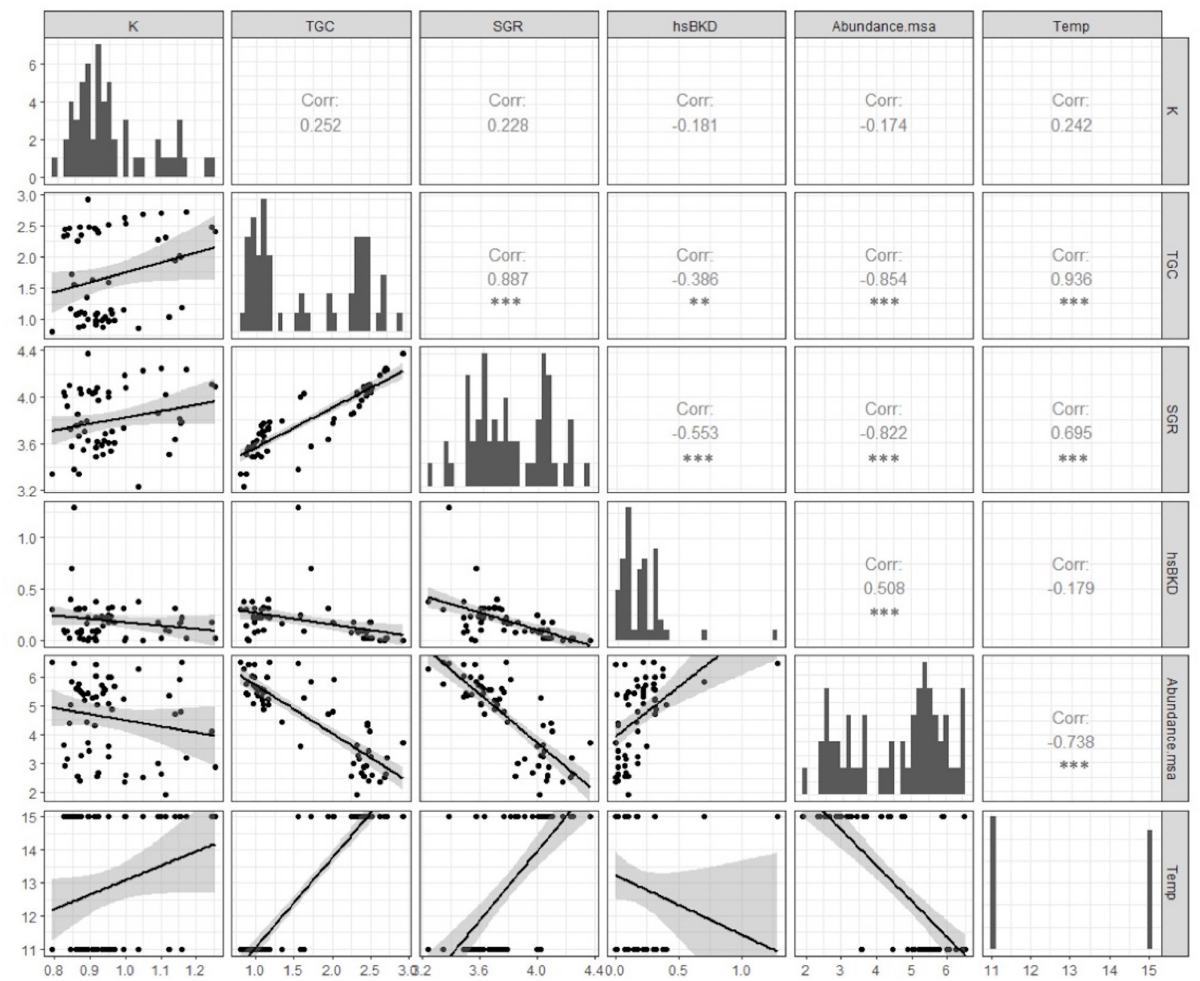
Pearson correlation coefficient (*r*) and *p*-value (*p*) between the abundance of msa transcripts, BKD histoscore and productive growth variables (SGR and TGC) in Atlantic salmon pre-smolts infected with *R. salmoninarum* (**p < 0.01, ***p < 0.001). The abundance of msa of *R. salmoninarum* induced a significant reduction in the key productive indicators in infected fish at 11 and 15°C. Additionally, infected fish shows a significant high level of association between productive indicators.

Tissue damage was relatively mild in both groups of infected fish compared to uninfected control fish (hsBKD <1.0), but significantly higher hsBKD was observed in fish infected at 11°C than at 15°C. However, we do not know if the lower water temperature modulated the presentation time and/or the severity of kidney damage. The most frequent microscopic findings in the kidneys were hematopoietic tissue hyperplasia, melano-macrophage centers hyperplasia, granulomas BKD-like and proliferative glomerulonephritis (Figure 2). The abundance of msa transcripts in the kidneys was significantly positively correlated with the histopathological lesions expressed as the hsBKD (Figure 3).

### *R. salmoninarum* Significantly Deteriorates Growth Indicators in Surviving Fish Exposed to Lower Water Temperatures

*R. salmoninarum* infection induced a significant reduction in the key productive indicators in infected fish at 11 and 15°C (Figure 3). Moreover, infected fish showed a significant high level of association between productive indicators (Figure 3). The abundance of msa transcripts showed a more significant association with the condition factor, SGR and TGC than the histoscore BKD (Figure 3). Therefore, fish growth would be modulated by the relative abundance of *R. salmoninarum* rather than cell and tissue damage.

### *R. salmoninarum* Induces Anemia and Impaired Renal Function in Surviving Fish Exposed to Lower Water Temperatures

**Figure 4:**
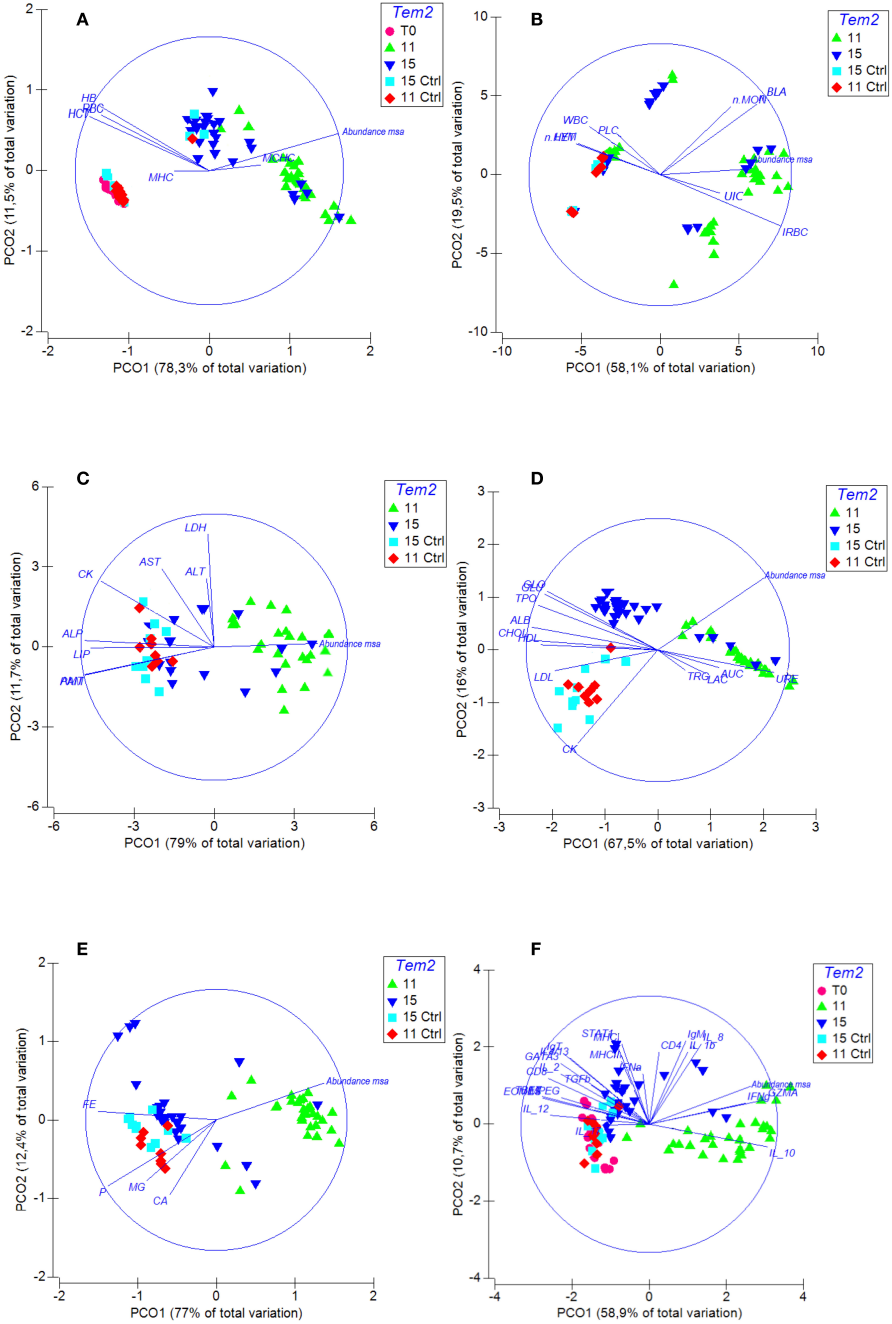
Spatial sorting of the different biomarkers of the immunopathological response of fish challenged with *R. salmoninarum* using a principal coordinate analysis (PCO). Euclidean distance matrices were used before standardization by log (x+1) for all data and independent for each group of variables. The PCOs were clustered into 5 groups: **(A)** Erythrogram. The abundance of msa transcripts of R. salmoninarum generated anemia in infected fish at 11°C compared to infected fish at a higher water temperature (HCT, RCR, HG, IRBC). **(B)** Leukogram and differential leukocyte count. PCO analysis shows that the abundance of msa transcripts modulated a milder inflammatory response in infected fish at 15°C compared to infected fish at 11°C. The fish infected at 11°C shows monocytosis and lymphopenia. **(C)** Plasma enzymes. Significant decreases were observed in the plasma concentration of different enzymes which are all indicators of poor nutritional condition during infection (AMI, PAM, LIP, ALP). **(D)** Plasma substrates: The infected fish at 11°C shows an increase in the serum concentrations of urea and creatinine and a decrease in the serum concentrations of total protein and albumin. Significant decreases were observed in the plasma concentration of glucose, cholesterol, HDL and LDL, which are all indicators of chronic inflammatory process and poor nutritional condition during infection. **(E)** Plasma minerals. The infected fish at 11°C shows an increase in the serum concentrations of iron. **(F)** Expression of genes involved in the immune response. PCO analysis shows that the cell-mediated immune response is downregulated more severely in infected fish at 11°C. Temp, temperature; Ctrl, control.

The infected fish at 11°C showed an increase in the serum concentrations of urea and creatinine and a decrease in the serum concentrations of total protein and albumin (Table 3). However, the concentrations of the same biomarkers in infected fish at 15°C were within the normal range. These biomarkers are indicators of kidney damage, and alterations in their levels could be linked to the macroscopic and microscopic lesions observed in the kidney that were associated with a high abundance of msa transcripts. Moreover, significant decreases were observed in the plasma concentration of glucose, cholesterol, HDL, LDL, and amylase, which are all indicators of kidney damage, chronic inflammatory process and poor nutritional condition during infection (Table 3). The increase in mineral levels, such as magnesium, phosphorus and calcium, are associated with kidney damage. The PCO analysis showed that the abundance of msa transcripts significantly changed the renal function and the overall condition of infected fish at 11°C compared to infected fish at 15°C (Figure 4).

Moreover, fish infected at 11°C showed a significant decrease in the total red blood cell count and hematocrit and hemoglobin concentration as well as a significant increase in the immature erythrocyte count (Table 3). On the other hand, these biomarkers were within the normal range in infected fish at 15°C. The PCO analysis showed that the abundance of msa transcripts of *R. salmoninarum* generated anemia in infected fish at 11°C compared to infected fish at a higher water temperature and uninfected fish (Figure 4). These findings are associated with the significantly high concentration of plasma iron in the same fish.

In contrast, the total white blood cells count was pathologically increased in infected fish at 15°C and showed significant differences with that of fish infected at 11°C, which showed a normal average count that was close to the maximum range. PCO analysis also showed that the abundance of msa transcripts modulated a milder inflammatory response in infected fish at 15°C compared to infected fish at 11°C and uninfected fish (Figure 4). The fish infected at 11°C showed monocytosis and lymphopenia.

### *R. salmoninarum* Induces a Significantly Greater Downregulation of the Cell-Mediated Immune Response Genes in Surviving Fish Exposed to Lower Water Temperatures

**Figure 5:**
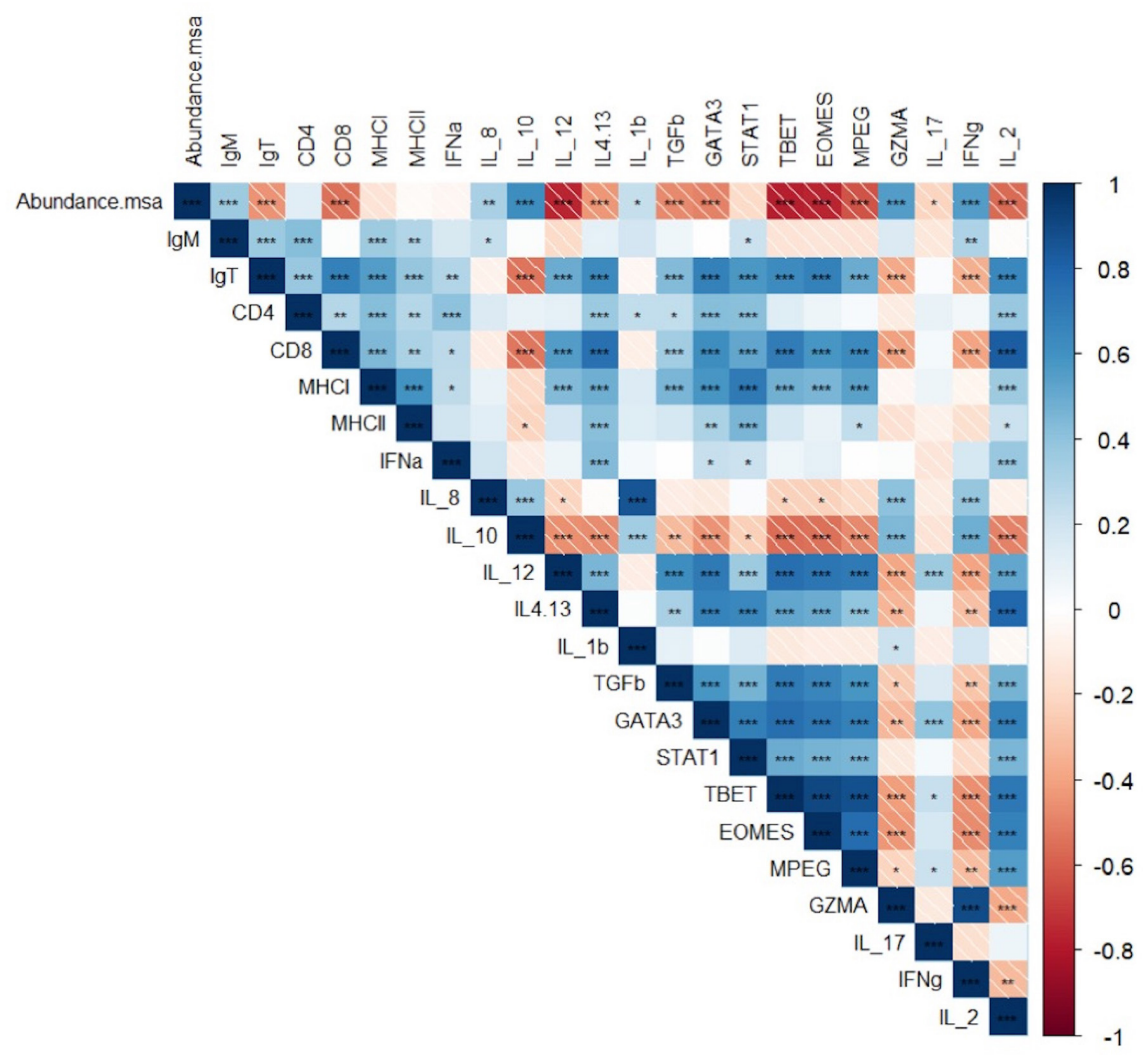
Pearson correlation coefficient (*r*) and *p*-value (*p*) between between the abundance of msa transcripts and the expression of target immune genes and association between the expression of each gene related to the innate and adaptive immune responses in the head kidney of Atlantic salmon pre-smolts infected with *R. salmoninarum* (*p < 0.05, **p < 0.01, ***p < 0.001).

In the late stage of infection, the abundance of msa transcripts of *R. salmoninarum* in the surviving fish from both groups induced a chronic pro-inflammatory response based on the significant overexpression of the transcripts of *IL-8* and *IFN*γ (Figure 5). At the same time, downregulation of Th2 cytokines, such as *IL4/IL13*, as well as *IL10* and *TGF*β, which are primarily associated with tissue repair and extracellular matrix composition, was observed during the late stage of infection, especially in infected fish at 11°C (Figure 5).

The abundance of msa of *R. salmoninarum* promoted the significant decreased the expression of the *T-bet* and *Eomes* transcription factors, cytokines *IL-12* and *IL-2, CD8*, and *perforin 2* (Table 3, Figure 5). Moreover, the load of *R. salmoninarum* showed a significant positive correlation with the downregulation of *IFN*γ*, Eomes, Tbet, GATA3, IL-2, IL-12, CD8*, and *perforin* (Table 3, Figure 5). Hence, *R. salmoninarum* did not induce an immune response mediated by CD8^+^ T-cells in infected fish at 11 or 15°C. The PCO analysis showed that the adaptive cell-mediated immune response was more severely downregulated in infected fish at 11°C (Figure 4).

Nevertheless, the infected fish at 15°C showed overexpression of *STAT1, MHCII, CD4, IgT*, and *IgM* transcripts, suggesting that the infection triggered a CD4^+^ T-cells response and a humoral response. However, this pattern of expression of genes related to humoral immunity was not observed in the infected fish at 11°C (Figure 5). Therefore, the best humoral response in infected fish at higher temperatures was not correlated with better protection because no significant differences were observed in the survival rates between both groups of fish. These results are unexpected in a fish infected with an intracellular bacterium and could partly explain the relative field efficacy of vaccines, although this supposition requires further investigation.

## Discussion

Understanding the pathophysiological mechanisms underlying the interaction *in vivo* of *R. salmoninarum* with its host, in this case, the Atlantic salmon, would allow for the development of alternative therapies for the treatment, prevention and control of BKD. We used different immunopathological biomarkers of infection with *R. salmoninarum* to better understand the nature of the host-pathogen interaction during the late stage of infection in Atlantic salmon maintained at different water temperatures. To our knowledge, previous studies have not examined late gene expression associated with the immune response against *R. salmoninarum* in surviving pre-smolts of Atlantic salmon, although previous studies have reported some aspects of the early immunopathological response of rainbow trout (16) and Chinook salmon challenged with *R. salmoninarum* (12), vaccinated Atlantic salmon parr (20) and vaccinated fish, although only *R. salmoninarum* agglutinating antibodies were used (20, 30, 31).

Although several environmental factors influence infectious disease dynamics in aquaculture, water temperature is considered one of the most important because it can determine pathogen and fish biology modulations (12–14). Our results confirmed that there was no significant relationship between water temperature and mean survival rate, although a higher probability of death and delay the mortality curve was observed in infected fish at 11°C than at 15°C after 35 days post-infection. These results are consistent with those reported by Purcell et al. (12), who showed that cold water contributes to greater BKD progression in Chinook salmon and the concomitant increase in bacterial shedding could contribute to greater horizontal transmission of the bacterium. Also, our findings are consistent with those described by Sanders et al. (15), who showed that lower temperatures resulted in higher mortality rates and longer mean times to mortality. However, our findings were not fully consistent with those described by Jones et al. (13), who showed that higher temperatures resulted in higher mortality rates.

The fish infected with *R. salmoninarum* and kept at 11 or 15°C had a mean survival of 21.5 and 20.1%, respectively. A survival challenge without fish sampling was conducted in Chinook salmon by Purcell et al. (32), and survival was 41% in the Wisconsin (WI) stock and 21% in the Washington (WA) stock. However, our results in Atlantic salmon were not consistent with the average survival rates of 61.3 and 79.8% later reported in Chinook salmon held at 8 and 12°C, respectively (12). One of the limitations of our study was that a low temperature between 6 and 8°C, which is closer to the BKD field conditions, could not be obtained.

Our current research is focused on applying the results of the present study as potential immunopathological biomarkers of a resistance phenotype for BKD to populations of 30 different Atlantic salmon families. Our preliminary results show that there is a clearly higher level of resistance of some families to BKD compared to others at 11°C (range of survival rate between 0 and 76%) (data not yet published). Evolutionary theory predicts that populations with sufficient genetic variation will show adaptive responses to pathogen pressure (33); however, WI and WA Chinook salmon groups challenged with *R. salmoninarum* at 14 and 9°C showed that although the warmer temperature accelerated the time of death in both populations, there was no evidence of phenotypic plasticity or a genotype interaction per environment interaction (14). High h^2^ estimates for BKD susceptibility in the WI population, combined with a lack of phenotypic plasticity, predicts that future adaptive gains in BKD resistance are still possible and that these adaptive gains would be stable under the temperature range evaluated (14). Results from the genome-wide association (GWA) studies indicate that BKD resistance has a polygenic genetic architecture with the largest single nucleotide polymorphism (SNP) explaining only 5.3% of the phenotypic variance (34). However, polygenic traits are often not improved greatly through selection based solely on a few significant markers due to the small proportion of variance explained by a single marker (35).

Mortality and survival rates are not the only outcome from *R. salmoninarum* infection. The infected fish at 11°C showed more severe clinical signs and pathological lesions than the infected fish at 15°C, a finding that may be associated with the expression of different virulence factors in the different water temperatures. A microscopic pathology analysis of BKD shows a chronic granulomatous inflammatory response to encapsulate the pathogen (4, 5). Typically, there is extensive tissue damage, a strong cell-mediated response, macrophage proliferation and activation, and probably the deposition of immune complexes in tissues (16, 17, 36). The major soluble antigen of *R. salmoninarum*, p57 (36–41), is produced in the tissues of infected fish, and it may have an important role in the chronic stimulation of TNFα, which could be expected to assist the chronic inflammatory pathology of BKD (16).

Although both groups of fish exhibited similar types of macroscopic kidney responses, the infected fish at 11°C showed a significantly higher BKD histological score, at least for the time period examined and with the infection metrics employed in this study. Purcell et al. (12) showed that changes in water temperature influence disease progression and the elimination of bacteria in Chinook salmon infected with *R. salmoninarum*. Fish held at 8°C showed the highest mortality rate and a higher renal abundance of *msa* transcripts in relation to fish held at 12°C, while fish held at 15°C showed little evidence of the progression of *R. salmoninarum* infection in relation to the group at 12°C.

*R. salmoninarum* produces abundant quantities of an extracellular, 57-kDa protein called p57 or major soluble antigen is widely known to be an important virulence factor (37, 38). The relative quantification of the abundance of *msa* transcripts in kidneys is expressed as the number of copies of the *msa* gene of *R. salmoninarum* in infected fish compared with that in the non-infected control group. The late stage of infection was characterized by a significantly greater amount of *msa* transcripts in the kidney of infected fish at 11°C than at 15°C. These results could indicate that the infected fish at 15°C appear to have been more successful at controlling the infection, although better protection was not observed because no significant differences were observed in the survival rates between both groups of fish.

Anemia and lymphopenia observed in infected fish at 11°C could be associated with the increased abundance of *msa* transcripts because this virulence factor shows several properties including the ability to bind salmonid erythrocytes (42), hemagglutinate mammalian erythrocytes (37, 39) agglutinate the leucocytes of various salmonid species (43), and adhere to Chinook salmon leucocytes (41). The lymphopenia observed in our study could also determine the typical immunosuppression process described in fish with BKD (43), making these fish more susceptible to opportunistic pathogens.

In salmonids, the development of semiquantitative histological indicators or histoscores has been described for SRS (44), heart disease (HSMI and CMS) (45), pancreas disease (PD) (46) and gill alterations (47), although there are no reports of histoscores for BKD. The severity of the microscopic kidney response expressed as hsBKD showed a significant positive correlation with bacterial growth, which was expressed as the *msa* transcripts of *R. salmoninarum*, and these results are consistent with previous descriptions of SRS pathogenesis in Atlantic salmon (44).

The increased serum level of UAC, CRE and minerals observed in this study indicates significant kidney damage, which is confirmed by the presence of microscopic lesions that suggest important alterations in kidney function. These results are consistent with the increase in CRE reported in sockeye salmon with BKD (48), Atlantic salmon infected with *P. salmonis* (44) and rainbow trout infected with *Serratia liquefaciens* (49).

The decrease in the level of TOP and ALB in fish infected with *R. salmoninarum* could be explained by a reduction in protein synthesis due to liver damage and by the higher excretion of protein or protein loss associated with kidney damage. Protein synthesis can decrease during infection due to the preferential synthesis of specific proteins, and albumin may act as an acute-phase negative protein (50). These results are consistent with reports in sockeye salmon infected with *R. salmoninarum* (48), Atlantic salmon infected with *P. salmonis* (44), tilapia, *Oreochromis* sp. infected with *Edwardsiella tarda* (51) and Salvelinus fontinalis species infected with *Flavobacterium columnare* (52). In addition, the decrease in the concentration of plasma substrates related to the general state of the fish was based on a decrease in the productive indicators of growth.

An effective antigen-specific CD8^+^ T-cells response is essential for immune protection against the dominant epitopes of intracellular bacteria and promotes the effective control of these organisms (53). Our results suggest that the abundance of msa transcripts of *R. salmoninarum* showed a significant positive correlation with the downregulation of *IFN*γ*, Eomes, Tbet, GATA3, IL-2, IL-12, CD8*, and *perforin*; consequently, *R. salmoninarum* did not induce an immune response mediated by CD8^+^ T-cells in infected fish at 11 or 15°C. These results showed the modulation of the cellular and humoral immune response based on the expression of genes associated with immune response of Atlantic salmon head kidney intraperitoneally infected with *R salmoninarum*, but we must consider that the transcriptomic approach alone is unable to draw conclusions about how bacterial infections affect activation/suppression of CD4 or CD8 T-cells function. Therefore, new studies at protein expression and activity level, immunological reagents and cytotoxic and/or helper T cell clones are essential for further development in the fish immunology.

All T cells possess a T cell receptor (TCR) by which they recognize peptide presented by MHC, along with CD3 and co-stimulatory, e.g., CD28, and co-inhibitory, e.g., CTLA-4, surface molecules (54). T cell associated genes and their encoded proteins with T cell activity in fish, e.g., surface markers, cytokines and transcriptional factors, have been well-documented (55). The presence of cytotoxic T cells (CTLs) and Th cells in fish have been identified as CD8^+^ and CD4^+^ T-cells, respectively using monoclonal antibodies (mAbs) (56–58). CD4 and CD8 molecules are expressed not only on T cells but also other cell types, e.g., CD4-1 in melano-macrophages in channel catfish (59). Thereby, multiple markers should be used for the true identification of T cells.

*R. salmoninarum* induced a significantly greater downregulation of the cell-mediated immune response genes in surviving fish exposed to lower water temperatures. This response could be due to a suppressed host response directly related to the lower water temperature and/or associated with a delayed host response related to the lower water temperature (60). However, acute infections may be resolved as a result of a temperature-dependent T-cell response, although carriers and latency are common (61). As reported for other important intracellular fish bacteria such as *Francisella noatunensis* (62), *P. salmonis* (26, 27, 63) and *Edwardsiella tarda* (64, 65), *R. salmoninarum* would require stimulation of cell-mediated immunity, although the mechanisms of this process in these species of bacteria remain poorly understood and require further investigation.

Yamasaki et al. (64) reported the important role of cellular immunity rather than humoral immunity against *E. tarda* infection in ginbuna cross carp. Bacterial clearance in the kidney and spleen was observed after elevated cytotoxic activity of CTLs and increased numbers of CD8α^+^ T-cells. However, *E. tarda*-specific antibody titers did not increase until after bacterial clearance, indicating that induction of humoral immunity would be too late to provide protection against infection. Rozas-Serri et al. (26) showed that *P. salmonis* induces the inflammatory and IFN-mediated response, modulation of Th1 polarization, reduced antigen processing and presentation, modulation of the evasion of the immune response mediated by CD8^+^ T-cells and promotion of the CD4^+^ T-cell response during the late stage of infection as a mechanism to escape host defenses. Additionally, Rozas-Serri et al. (63) showed several central signatures following infection with *P. salmonis* in Atlantic salmon, including positive regulation of *DC-SIGN* and *TLR5* signaling, which converged at the *NF-kB* level to modulate the pro-inflammatory cytokine response. *P. salmonis* induced an IFN-inducible response, e.g., *IRF-1* and *GBP-1*, but inhibited the humoral and cell-mediated immune responses.

Yamasaki et al. (65) demonstrated the importance of cell-mediated immunity against *E. tarda* infection using vaccine trials comparing the effects of live vs. formalin-killed bacteria. Live cell-vaccinated fish showed high survival rates, high *IFN*γ and *T-bet* gene expression levels, and increased CTLs. On contrary, all bacterin-vaccinated fish died following *E. tarda* infection and induced high *IL4/13a* and *IL-10* expression levels, whereas Th1-like responses were suppressed. Similarly, Rozas-Serri et al. (27) showed that a bacterin *P. salmonis* vaccinated-fish exhibited *MHCI, MHCII*, and *CD4* overexpression but a significant downregulation of *CD8b* and *IgM*, suggesting that the formalin-killed bacteria promoted the CD4^+^ T-cell response but did not induce an immune response mediated by CD8^+^ T cells or a humoral response.

Our work shows that infected fish at 15°C presented the overexpression of *STAT1, MHCII, CD4, IgT*, and *IgM* transcripts, suggesting that the infection triggered a CD4^+^ T-cells response and a humoral response. However, this pattern of expression of genes related to humoral immunity was not observed in infected fish at 11°C. Salmon infected with *R. salmoninarum* produce an antibody response, although it is not necessarily correlated with protection (31, 66). Other studies have shown that p57 suppresses antibody responses and macrophage respiratory burst and renders immunized animals more susceptible to BKD (16, 38, 40).

Underexpression of Th2 cytokines, such as *IL4/IL13*, as well as *IL10* and *TGF*β, which are primarily associated with tissue repair and extracellular matrix composition, was observed during the late stage of infection, especially in infected fish at 11°C (67, 68). In addition, our results show late underexpression of *IL-1*β, which could induce long-term suppression of cytokine production, phagocyte function, and lymphocyte proliferation and activation, including T-cell-dependent antibody production (69–71). The downregulation of these cytokines during the late stage of infection may affect each of these pathways and facilitate the survival of *R. salmoninarum*.

The chronic higher levels of IFNγ may be implicated in the pathology of BKD and the host-mediated destruction of kidney tissues (16). These results would suggest how *R. salmoninarum* could suppress the host immune response and suggest that the immune mechanisms for the containment of *R. salmoninarum* infections rely on cell-mediated immune response-dependent pathways. Therefore, prolonged stimulation of IFNγ observed in infected fish at 11°C may contribute to the chronic inflammatory pathology of BKD. There is evidence that *R. salmoninarum* has mechanisms to evade detection by the host's immune system (16), which is also observed for other intracellular bacteria, such as *P. salmonis* (26, 27, 44, 63). The identification of CD4^+^ and CD8^+^ T-cell antigens and development of a method to stimulate lasting immunity represent main challenges.

Our results show that the expression patterns of genes related to the humoral and cell-mediated immune response in Atlantic salmon infected with *R. salmoninarum* at 11 and 15°C were very similar; however, *R. salmoninarum* induced a significantly greater downregulation of the adaptive immune response genes at the lower water temperature. A constant suppression of the adaptive immune system is observed in response to colder temperatures, which could be associated with detrimental effects of low temperatures on the innate immune system (60). However, better protection was not observed because the mean survival rates did not show differences between the water temperature groups, although a higher probability of death was observed in infected fish at 11°C than at 15°C after 35 dpi. These results are similar to those reported in different populations of Chinook salmon by Metzger et al. (17), who demonstrated that bacterial load levels positively regulated pro-inflammatory genes expression in fish from both groups despite the higher mortality in the more susceptible Green River stock.

Genetic variability between families of Atlantic salmon and/or the use of different challenge models could show different results than those described here, so further investigation is required. Challenge models using baths and cohabitation more faithfully represent the conditions of natural exposure compared with the i.p. infection (72, 73). This method would bypass any first-barrier defense mechanism that prevents the disease because bacteria are not transferred from the environment to the fish. Our experience with *P. salmonis*, another intracellular bacterium that is of great economic relevance in Chile and modulates the immune response of Atlantic salmon using the same strategy described here for *R. salmoninarum*, indicates that evaluating the host-pathogen interaction using an i.p. challenge model generates different results compared with a cohabitation model (26, 27, 44, 63). Thus, it would be interesting to study the pathogenesis and immune response against BKD and vaccines to BKD using a cohabitation model (73). Thereby, it is important to acknowledge the potential value in performing more comprehensive studies that can strengthen our understanding of the impact of low water temperatures on host-pathogen systems.

Taken together, our results show differences in the immunopathological response of pre-smolts of Atlantic salmon infected with *R. salmoninarum* at different water temperatures, but this differential response could be related to a suppressed host response and/or a delayed host response because they were exposed to a lower water temperature. The virulence of pathogens is affected by water temperature (74), although it may be difficult to separate these effects from those of the host immune system. The effect of water temperature on fish immune systems thus varies depending upon the duration and magnitude of the temperature change. In general, lower temperatures lead to a shutdown or slowing of immune response mechanisms, which is generally reversible upon return to warmer temperatures (60).

At 55 days after infection, the surviving fish kept at 11 and 15°C were carriers of *msa* transcripts of *R. salmoninarum*, which would have downregulated the humoral and cell-mediated adaptive immune genes and persisted chronically in the kidneys. Hence, these carrier fish may have a higher risk of recurrent outbreaks under stress conditions, such as during the transfer of Atlantic salmon smolts to seawater farms with low temperatures. Therefore, more studies are needed to determine the molecular mechanisms underlying the regulation of virulence genes expression in response to temperature in *R. salmoninarum*.

These results provide valuable information on the modulation of the late adaptive immune response in fish after *R. salmoninarum* infection, and we hope that they will be useful for optimizing the health and productive management of farmed fish and designing vaccines to provide long-term protection in fish. In addition, our results supported the identification of 33 immunopathological biomarkers for potential application in the search for a resistance phenotype for BKD, and eight of these genes are related specifically to the adaptive cell-mediated immune response. Finally, our current research is focused on applying the results of the present study as potential immunopathological biomarkers of a resistance phenotype for BKD to populations of 30 different Atlantic salmon families.

## Data Availability Statement

Publicly available datasets were analyzed in this study. Requests to access the datasets should be directed to Marco Rozas-Serri at marco.rozas@pathovet.cl.

## Ethics Statement

The animal study was reviewed and approved by Elanco's Animal Welfare Program which covers all of Elanco's animal facilities worldwide and ensures the care and use of all animals. Therefore, the study that was completed at the Aquarium Facility in Puerto Varas was reviewed and approved by the Elanco Global Ethical Committee (Institutional Animal Care and Use Committee). Written informed consent was obtained from the owners for the participation of their animals in this study.

## Author Contributions

MR-S designed and directed the experiment, fully analyzed the results, and wrote the manuscript. CL designed the experiment and partially analyzed the results. RC, RI, VJ, CO, and DC designed and validated the histoscore BKD and performed the processing and histopathological diagnosis. JV, AM, LM, and AP validated the molecular analyses and executed the RT-PCRs analysis. RW and CN performed the hematological processing and diagnosis and blood biochemical profiles. JG, PM, and FS carried out the sampling of the fish and taking their respective tissues at the hatchery for the subsequent processing and diagnosis in the laboratory. All authors contributed to the article and approved the submitted version.

## Conflict of Interest

MR-S, RC, RI, JV, AM, LM, VJ, DC, CO, RW, CN, JG, PM, AP, and CS are employed by Laboratorio Pethovet Ltda. MR-S is also employed by Newenko Group SpA. CL and FS are employed by Hendrix Genetics Aquaculture S.A. The authors declare that this study received funding from Hendrix Genetics Aquaculture. The funder had the following involvement in the study: design and decision to submit it for publication.
